# Application of metagenomic next-generation sequencing in the diagnosis of *Bartonella* neuroretinitis: a case report and literature review

**DOI:** 10.1186/s12348-024-00387-0

**Published:** 2024-04-19

**Authors:** Pengcheng Li, Zhuyun Qian, Yong Tao

**Affiliations:** 1 Department of Ophthalmology, Union Hospital, Tongji Medical College, Huazhong University of Science and Technology, Hubei, China; 2 Beijing GIANTMED Medical Diagnostics Lab, Beijing, China; 3 Department of Ophthalmology, Beijing Chaoyang Hospital, Capital Medical University, No. 8, South Road of Worker’s Stadium Chaoyang District, Beijing, 100020 China

**Keywords:** Neuroretinitis, *Bartonella henselae*, cat-scratch disease, Metagenomic next-generation sequencing, Case report

## Abstract

**Background:**

Cat-scratch disease (CSD) is caused by *Bartonella henselae* infection. In atypical cases of CSD, pathogen determination is challenging. We report a case of *Bartonella* neuroretinitis with neither a clear history of scratches nor typical general symptoms. The diagnosis was made using metagenomic next-generation sequencing (mNGS), a high-throughput sequencing technology.

**Case presentation:**

A female patient presented to the ophthalmologist with complaint of blurred vision in her right eye. Although with history of raising a cat, she reported no clear history of scratches or typical general symptoms, except a fever of unknown origin which resolved spontaneously. The best corrected visual acuity (BCVA) of the right eye was count fingers. Fundus examination showed optic disc oedema, macular exudates and inferior exudative retinal detachment. Laboratory examination results showed increased value of serum C-reactive protein and erythrocyte sedimentation rate. Ocular involvement of toxoplasmosis, syphilis and tuberculosis were excluded. To identify the possible causative pathogen of the disease, mNGS of aqueous humour sample was performed and 521 reads of *B. henselae* were identified. Serological test results further showed a positive immunoglobulin G (IgG) titre of 1:64. Taking the contact history, clinical manifestations, mNGS and serological results into consideration, the diagnosis of *Bartonella* neuroretinitis (ocular CSD) was made. After appropriate treatment, the BCVA of the right eye improved to 20/25 in the last follow-up. Fundus examination showed a normal optic disc and macula, and the exudates had reduced.

**Conclusion:**

mNGS, a fast and unbiased method, can be used to detect *B. henselae* (if present) in intraocular fluid samples.; however, the results should be interpreted together with the clinical symptoms and other auxiliary test results.

## Background

*Bartonella henselae* is a small, fastidious, Gram-negative intracellular bacillus that can cause cat-scratch disease (CSD). Initial erythematous papules at the infection site and regional lymphadenopathy are common clinical manifestations of CSD, while in more severe cases, atypical symptoms may occur, such as osteitis, myalgia, arthropathy, bacteraemia, endocarditis, encephalitis, hepatosplenic abscesses, bacillary angiomatosis, ocular involvement and fever of unknown origin [1, 2]. Neuroretinitis, which is characterised by optic disc oedema and subsequent formation of a macular star, is one of the most common forms of ocular CSD [3].

Diagnosis of *Bartonella* neuroretinitis is based on a history of contact with a cat, typical ocular signs, systemic symptoms and positive laboratory examination results. The indirect fluorescent antibody (IFA) test is the most reliable and widely used laboratory test for *Bartonella* neuroretinitis diagnosis. Previous study demonstrated a serological positive rate of 9.68% in China, which ranged with age (0%-30.43%) and region (urban residents:0.95% and rural residents:10.41%) [4]. Nucleic acid tests, such as the polymerase chain reaction (PCR), are not routinely performed with intraocular fluid samples [5]. However, considerable developments in metagenomic next-generation sequencing (mNGS) over the past five years have made it viable to utilise this unbiased, high-throughput sequencing technology in the diagnosis of suspected intraocular infections.

Here, we report a case of *Bartonella* neuroretinitis in which the causative pathogen was identified using mNGS. We have also reviewed previous studies that focused on the application of mNGS in the diagnosis of CSD.

### Case presentation

A 37-year-old female patient presented to the ophthalmology clinic at Union Hospital on 16 December 2021 with a complaint of blurred vision in her right eye for one week. She had a fever of unknown origin five days before, which resolved spontaneously. Five days earlier, the patient was diagnosed with optic neuritis at a regional hospital and received oral corticosteroids, and her symptoms were well controlled. The patient reported a history of raising a cat at home and that she had not been scratched in the past few months. A general physical examination did not show any skin lesions or lymphadenopathy. The patient’s laboratory examination results showed normal complete blood count and autoimmune antibody values. However, increased serum C-reactive protein (CRP; 108 mg/L; normal = 0–5 mg/L) and erythrocyte sedimentation rate (ESR; 51 mm/h; normal = 0–20 mm/h) values were detected.

**Fig. 1:**
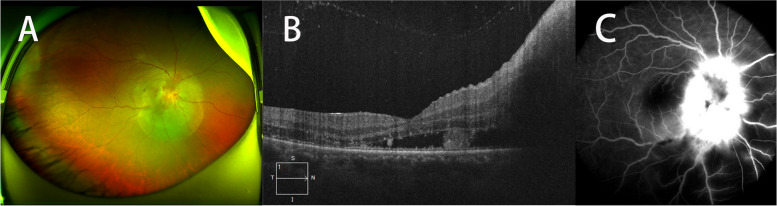
Wide-angle fundus photography showed optic disc oedema, macular exudates and inferior exudative retinal detachment (**A**). Macular OCT showed subretinal fluid with hyperreflective signals at the level of the outer plexiform layer and outer nuclear layer (**B**). FFA showed optic disc hyperfluorescence in the late phase (**C**)

The patient underwent detailed ophthalmic examinations, including best corrected visual acuity (BCVA), tonometry, slit-lamp examination, fundus examination, optical coherence tomography (OCT), fundus fluorescein angiography (FFA), visual field examination and visual evoked potential (VEP). No abnormal results were found in the left eye. In the right eye, the BCVA was count fingers, the intraocular pressure was normal and the anterior segment was quiet. Fundus examination showed optic disc oedema, macular exudates and inferior exudative retinal detachment. Macular OCT showed subretinal fluid with hyperreflective signals at the level of the outer plexiform layer and outer nuclear layer. FFA showed optic disc hyperfluorescence in the late phase (Fig. 1). Visual field examination showed that only an inferonasal island of vision remained, while VEP showed a prolonged implicit time and reduced P2 wave amplitude. Based on the clinical symptoms, ocular signs and results of the auxiliary examinations, the diagnosis of neuroretinitis in the right eye was made.

**Fig. 2:**
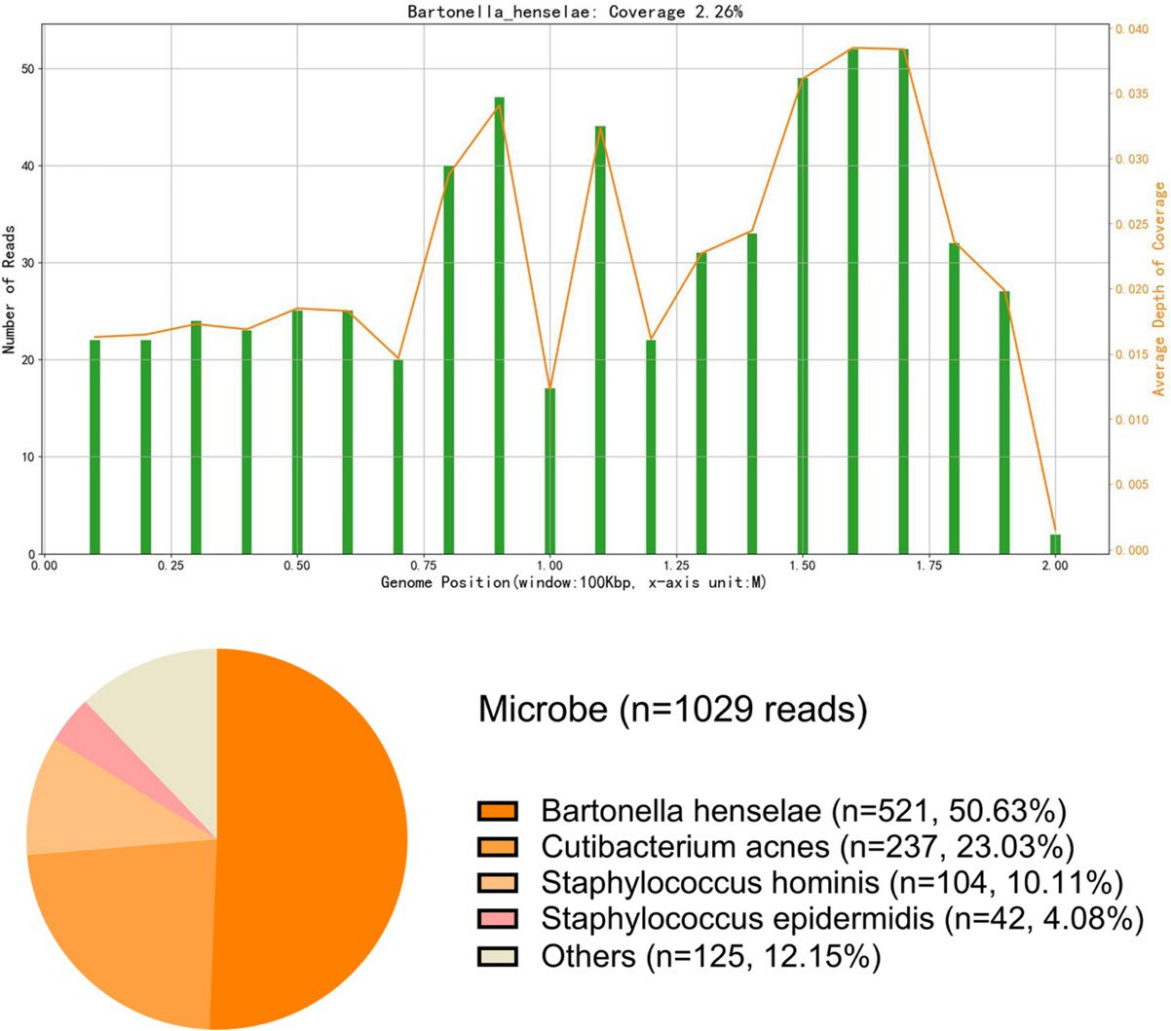
The mNGS results from the aqueous humour sample showed that the coverage of *Bartonella henselae* reads was 2.26% with an equal distribution. These reads accounted for the largest proportion of identified pathogen reads. In the upper figure, the reference data of Bartonella henselae genome (about 1.9M) were divided into 20 subunits (100Kbp). The number of reads aligned to each subunit and the average depth of coverage were presented in Y-axis

**Fig. 3:**
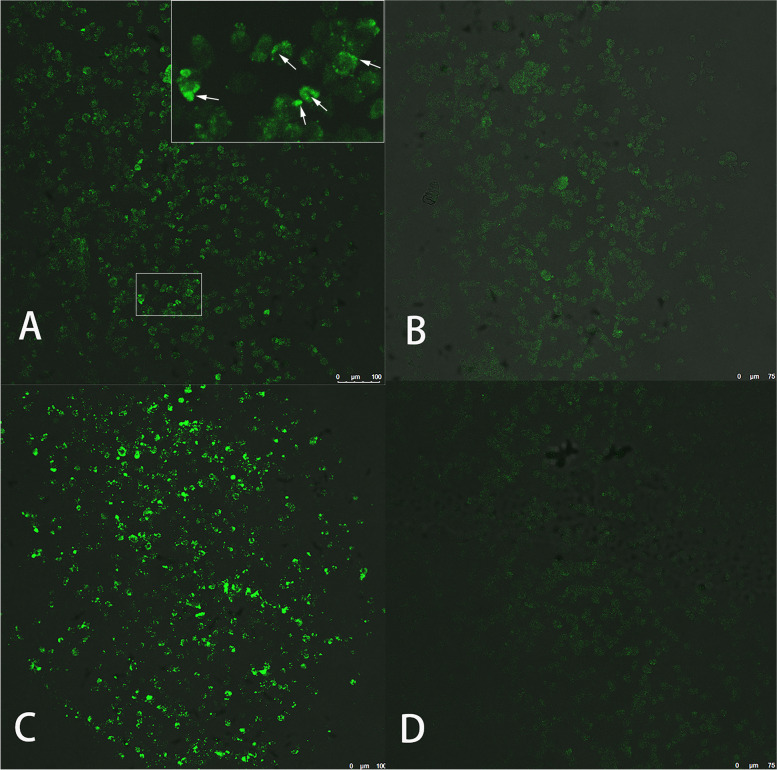
The results of the IFA test for *Bartonella henselae* showed moderate apple-green fluorescence at a titre of 1:64 (**A**) and no fluorescence at a titre of 1:256 (**B)**. The positive control and negative control are shown in (**C**) and (**D**), respectively

To identify the possible causative pathogen of the disease, serological tests for *Toxoplasma* and *Treponema* were conducted, and the results were negative. A T-SPOT.*TB* test also returned a negative result. Serological tests for Lyme borreliosis and rickettsia were not available. mNGS test was performed to screening for potential infectious pathogen. Considering the accessibility of different intraocular sample types and the lack of indication of pars plana vitrectomy, aqueous humour was used in the test procedure. With the patient’s consent, about 100 μL of aqueous humour was obtained through paracentesis performed at the 7 o’clock position of the cornea. The sample was sent to GiantMed Diagnostics (Beijing) for mNGS on the Nextseq 550 platform (75 base pair [bp] single-end reads; Illumina, San Diego, USA). A total of 42,469,925 reads were generated, and 39,640,698 reads were aligned with human sequences using SNAP software (version 2.0, Swift Biosciences™, Ann Arbor, MI, USA). In the downstream analysis, adapter, plasmid, duplicate, low-quality, low-complexity and short reads (< 35 bp) were filtered out and 5371 reads remained as the total reads of detected microbes. Reads associated with contaminating microbes from laboratory environment and engineered bacteria from the enzymes and buffer solutions used in the wet lab procedure were also removed. The remaining 1029 reads were then blasted against the Microbial Genomes database (ftp://ftp.ncbi.nlm.nih.gov/genomes/) using Burrows–Wheeler Alignment. The database consists of microbial genomes collected by the National Center for Biotechnology Information (NCBI) from more than 20,000 microorganisms, including 11,910 bacteria, 7,103 viruses, 1,046 fungi and 305 parasites. A total of 521 reads aligned uniquely with the *B. henselae* reference genome, with a genome coverage of 2.26% (Fig. 2). To verify the mNGS result, an IFA test of the serum sample for anti-*B. henselae* immunoglobulin G (IgG) antibody titers was performed. The test was performed according to the kit manufacturer's instructions (IF1300G, Focus Technologies, Cypress, CA) as described in the previous study [6]. The result of IFA test was positive, with a titre of 1:64 (Fig. 3). Test for anti-*B.hensenlae* immunoglobulin M (IgM) was not available in our hospital. Taking the contact history, clinical manifestations, mNGS and serological results into consideration, the diagnosis of *Bartonella* neuroretinitis (ocular CSD) was made.

**Fig. 4:**
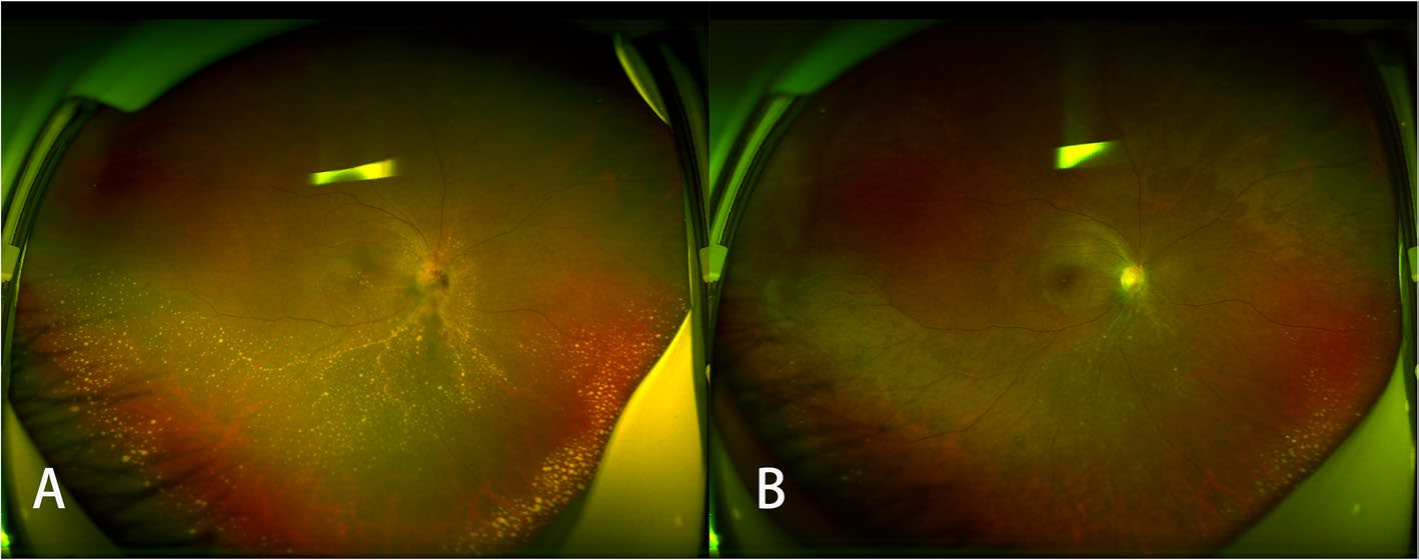
Wide-angle fundus photography showed lightened optic disc oedema, decreased macular exudates, absorption of subretinal fluid and massive exudates in the inferior part of the retina six weeks after the onset of disease (**A**), and less extensive exudates and a normal optic disc and macula at the end of the follow-up period (**B**)

After the diagnosis was made, the patient was treated with doxycycline (200 mg/day), rifampicin (600 mg/day) and azithromycin (250 mg/day), together with oral prednisolone (30 mg/day), which was gradually tapered down over six weeks. When the oral medications were discontinued, the patient’s BCVA was 20/60 (Snellen visual acuity chart). Fundus examination showed lightened optic disc oedema, decreased macular exudates and absorption of subretinal fluid. Massive exudates could still be observed in the inferior part of the retina. The last follow-up was six months after the onset of the disease. At that point, the BCVA of the right eye was 20/25. Fundus examination showed a normal optic disc and macula, and the exudates had reduced (Fig. 4).

## Discussion and conclusions

Infection with *B. henselae* can cause various ocular diseases and protean manifestations, including uveitis, neuroretinitis, multifocal retinitis, retinal vessel occlusion and Parinaud’s oculoglandular syndrome [7]. Neuroretinitis is the most common posterior segment complication in CSD [8]. In a patient with a clear contact history, typical general symptoms and ocular signs, it is not difficult to determine the causative pathogen of the disease. However, the patient in our case did not recall being scratched; neither did she have any notable general symptoms, except a transient fever. In this situation, it was essential to exclude other potential causes of the disease. The aetiologies of neuroretinitis include both noninfectious diseases (e.g. sarcoidosis, Behçet’s disease and systemic lupus erythematosus) and infectious diseases (e.g. rickettsioses, Lyme disease, toxoplasmosis, tuberculosis, syphilis and CSD). The normal serum autoimmune antibody levels and unilateral ocular involvement of this patient implied an infectious cause. In our hospital, laboratory tests for infectious diseases only covered a few pathogens (tests for Lyme borreliosis and rickettsia were not available). With the purpose of screening more possible pathogens in one test, mNGS was applied, and *B. henselae* was detected.

mNGS played a pivotal role in the diagnostic process of this case. This technology can simultaneously detect tens of thousands of pathogens in a limited sample volume, which makes it particularly valuable in the diagnosis of rare, atypical and complex infections [9]. To investigate the extent to which mNGS has been used to detect B. henselae, we performed a systematic PubMed search with the terms ‘metagenomic next-generation sequencing’, ‘cat-scratch disease’ and ‘Bartonella henselae’. Only reports published in English were included. A total of five studies were found that mention the application of mNGS in the diagnosis of CSD using different types of samples (Table 1), including lymph node tissue, tissue swabs, plasma, peripheral blood and cerebrospinal fluid [10–14]. Although the clinical manifestations, *B. henselae*-specific sequences identified using mNGS and genome coverage varied considerably among the studies, they all proved that mNGS could be of help if conventional tests did not yield a diagnosis in cases of complicated general infection. To our knowledge, this is the first report of the use of mNGS for the identification of *B. henselae* in an intraocular fluid sample.

**Table 1:** Summary of five cases with the application of mNGS in diagnosis of cat-scratch disease

Author (Ref)	Age (yr)/Sex	Contact with cats	Clinical manifestation	Sample type	B. henselae-specific sequences (reads)^a^	Genome coverage^b^	Other microbiology
Yang et al. [10]	48/Female	Yes	Intermittent fever, systemic rash, fatigue, anorexia, weight loss, shock and unconsciousness	Lymph node tissue	7182	13.94%	NA^c^
Wang et al. [11]	65/Male	NA	Fever, subcutaneous abcess	Tissue swab	305	0.9%	Culture negative
Patel et al. [12]	65/Male	Yes	Fever, shortness of breath and chest pain	Plasma	NA	NA	Culture negative, serology positive
Kassab et al. [13]	49/Male	No	Fever, tensionlike headache, nausea, and non-bloody emesis	Cerebrospinal fluid	22	0.13%	Culture negative, PCR negative, serology positive
Li et al. [14]	13/Male	Yes	Intermittent fever, headache, poor appetite and weight loss	Peripheral blood	4	NA	Culture negative

^a^The specific and only mapped sequence number to the pathogen genome

^b^The proportion of the sequence number covered the whole pathogen genome

^c^Not available

Zhu et al. [15] investigated the utility of mNGS for the identification of pathogens in cases of endophthalmitis and concluded that the positive identification rate was significantly higher when mNGS was used than culturing (88.89% vs. 27.78%). This proved the reliability of mNGS for pathogen detection in suspected intraocular infection. Also, in a study of the application of mNGS in infectious keratitis by Lalitha et al. [16], the sensitivity and the specificity were 62%-100% and 97% in bacterial keratitis, and were 65%-100% and 100% in fungal keratitis, respectively. However, interpretation of mNGS results should be performed with caution. Due to the high sensitivity and complexity of the methodology, contamination is almost inevitable. It is sometimes difficult to distinguish among colonising, background and actual pathogenic bacteria [15]. Hence, it is vital to analyse mNGS data together with the clinical manifestations and the results of other laboratory examinations. In our case, *B. henselae*, a well-known pathogen that causes neuroretinitis, was identified by mNGS. Although the result of serological test showed an IgG titer of 1:64, which did not underline that Bartonella was definitely causative for the neuroretinitis, we believed that the *B. henselae* was the most possible causative agent in this case with both molecular biological and serological evidence. In order to reduce the medical costs and to avoid unnecessary invasive procedure, the serological test was not repeated. However, a positive IgM titer or a higher IgG titer in a repeated test 10–14 days later would be helpful to make a more confirmed diagnosis. Besides the serological test, some molecular assay, such as targeted Bartonella PCR test or 16S-based molecular assay [17], could also be valuable to confirm the mNGS result in similar cases in the future.

In terms of treatment, the antibiotics doxycycline, rifampicin and azithromycin were selected for our patient. Considering the severe intraocular inflammation (i.e. the existence of inferior exudative retinal detachment), a corticosteroid was also prescribed. There is no consensus on the treatment modalities for ocular CSD [18]. However, doxycycline alone or in association with rifampicin and azithromycin is the typical first-line antibiotic treatment used; erythromycin, ciprofloxacin, trimethoprim-sulfamethoxazole and gentamicin are also used, but less commonly [8, 18, 19]. *Bartonella* neuroretinitis is a self-limiting disease in immunocompetent patients and usually has a favourable prognosis. In our case, optic disc oedema and macular exudates were still present six weeks after starting treatment and totally resolved within six months. The patient also recovered normal visual acuity.

In summary, we have reported a case of *Bartonella* neuroretinitis with neither a clear history of scratches nor typical symptoms. The diagnosis was based on the results obtained from mNGS. Our findings show that mNGS is a fast and unbiased method that can be used to detect *B. henselae* in intraocular fluid samples. However, mNGS results should be interpreted with caution, and the ultimate diagnosis of ocular CSD should be based on ocular symptoms, mNGS data and additional auxiliary and laboratory test results.

## Data Availability

All data generated or analyzed during this study are included in this published article.

## Abbreviations

CSD: Cat-scratch disease
IFA: Indirect fluorescent antibody
PCR: Polymerase chain reaction
mNGS: Metagenomic next-generation sequencing
BCVA: Best corrected visual acuity
OCT: Optical coherence tomography
FFA: Fundus fluorescein angiography
VEP: Visual evoked potential
IgG: Immunoglobulin G
IgM: Immunoglobulin M
